# Applications of SNAP‐tag technology in skin cancer therapy

**DOI:** 10.1002/hsr2.103

**Published:** 2019-01-08

**Authors:** Eden Rebecca Padayachee, Henry Ademola Adeola, Jennifer Catherine Van Wyk, Fleury Augustine Nsole Biteghe, Shivan Chetty, Nonhlanhla Patience Khumalo, Stefan Barth

**Affiliations:** ^1^ Department of Integrative Biomedical Sciences, Institute of Infectious Disease and Molecular Medicine, Faculty of Health Sciences University of Cape Town Cape Town South Africa; ^2^ The Hair and Skin Research Lab, Division of Dermatology, Department of Medicine, Faculty of Health Sciences University of Cape Town and Groote Schuur Hospital Cape Town South Africa

**Keywords:** antibody drug conjugates, benzylguanine, skin cancer, SNAP‐tag, targeted therapies

## Abstract

**Background:**

Cancer treatment in the 21st century has seen immense advances in optical imaging and immunotherapy. Significant progress has been made in the bioengineering and production of immunoconjugates to achieve the goal of specifically targeting tumors.

**Discussion:**

In the 21st century, antibody drug conjugates (ADCs) have been the focus of immunotherapeutic strategies in cancer. ADCs combine the unique targeting of monoclonal antibodies (mAbs) with the cancer killing ability of cytotoxic drugs. However, due to random conjugation methods of drug to antibody, ADCs are associated with poor antigen specificity and low cytotoxicity, resulting in a drug to antibody ratio (DAR) >1. This means that the cytotoxic drugs in ADCs are conjugated randomly to antibodies, by cysteine or lysine residues. This generates heterogeneous ADC populations with 0 to 8 drugs per an antibody, each with distinct pharmacokinetic, efficacy, and toxicity properties. Additionally, heterogeneity is created not only by different antibody to ligand ratios but also by different sites of conjugation. Hence, much effort has been made to find and establish antibody conjugation strategies that enable us to better control stoichiometry and site‐specificity. This includes utilizing protein self‐labeling tags as fusion partners to the original protein. Site‐specific conjugation is a significant characteristic of these engineered proteins. SNAP‐tag is one such engineered self‐labeling protein tag shown to have promising potential in cancer treatment. The SNAP‐tag is fused to an antibody of choice and covalently reacts specifically in a 1:1 ratio with benzylguanine (BG) substrates, eg, fluorophores or photosensitizers, to target skin cancer. This makes SNAP‐tag a versatile technique in optical imaging and photoimmunotherapy of skin cancer.

**Conclusion:**

SNAP‐tag technology has the potential to contribute greatly to a broad range of molecular oncological applications because it combines efficacious tumor targeting, minimized local and systemic toxicity, and noninvasive assessment of diagnostic/prognostic molecular biomarkers of cancer.

## 1 INTRODUCTION

### 1.1 Skin cancer

Skin cancer occurs as two main types, ie, non‐melanoma and melanoma. Non‐melanoma types include basal cell carcinoma (BCC) and squamous cell carcinoma (SCC), which are of keratinocyte origin, as well as Merkel cell carcinoma (MCC), sebaceous gland tumors, and malignant pilomatrixoma.^1^, ^2^ Melanoma types include superficial spreading melanoma (SSM), lentigo maligna melanoma (LMM), nodular melanoma (NM), acral lentiginous melanoma (ALM), mucosal melanoma, desmoplastic melanoma, and nevoid melanoma.^3^

In addition, non‐melanoma skin cancers (NMSC) can have more than one histological subtype, in which case they are referred to as mixed types.^4^ There is variability in the behavior of different types of skin cancer, as well as histopathological variants, depending on growth patterns.^4^ For example, BCC carcinomas grow slowly, with damage to surrounding tissue, but rarely spreads to vital structures, whereas SCC and melanomas are aggressive and are more likely to metastasize.^5^, ^6^ Solar and actinic keratitis, viral warts, and Bowen disease increase the risk of NMSC, while clinically atypical mole (CAM), giant congenital melanocytic nevi, and lentigo maligna have been shown to increase the risk of developing melanoma.^7^ The risk of developing skin cancer is also higher in people with poor immune function (such as HIV/AIDS and solid organ transplant patients)^8^, ^9^, ^10^ and people of fair skin color.^10^, ^11^, ^12^, ^13^, ^14^

**Figure 1:**
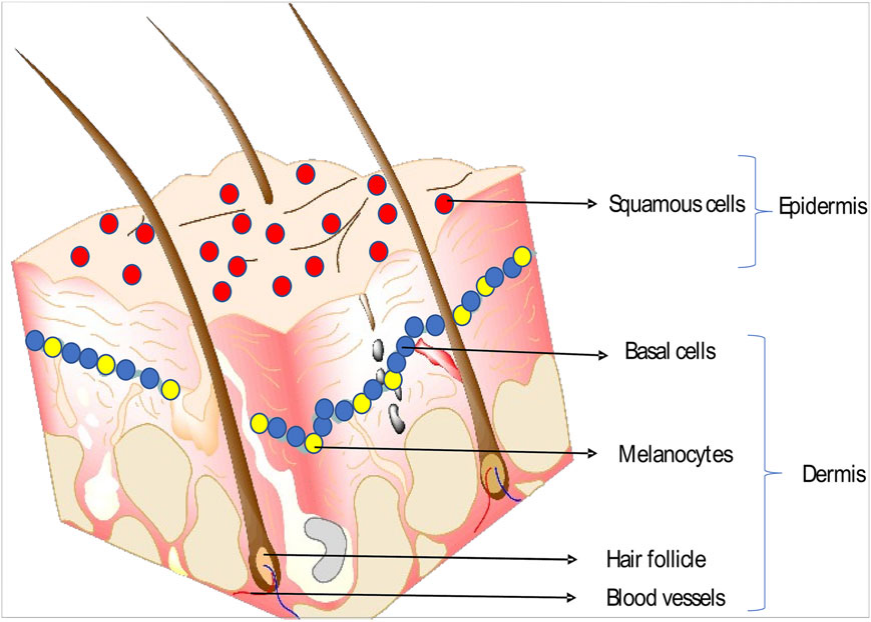
A sketch showing the squamous cells, melanocytes, and basal cells found in the epidermal layer of the skin. Ultraviolet (UV) light from the sun can damage the DNA in these skin cells and give rise to SCC, BCC, or melanoma

The observed increases in skin cancer rates are associated with several factors, including the fact that older populations are at higher risk of NMSC, and also increased occupational and recreational UV light exposure^15^, ^16^ (Figure 1). For instance, studies have shown that indoor tanning is associated with a significantly increased risk of BCC and SCC, with a higher risk with use in early life (<25 y).^17^ Each year in the United States, over 5.4 million cases of NMSC are treated in more than 3.3 million people.^18^ In 2017, it was estimated that 87 110 new cases of invasive melanoma were diagnosed in the United States and an estimated 9730 people were casualties of this aggressive type of skin cancer.^19^ The annual cost of treating skin cancers in the United States is estimated at $8.1 billion: about $4.8 billion for NMSC and $3.3 billion for melanoma.^20^

#### 1.1.1 Current skin cancer treatment

The use of conventional cancer therapies for skin cancers has been fraught with poor specificity in targeting the cancer cells, partly due to variations in surface receptor expressed by tumor cells.^21^ Surgical therapy may be used depending on the type and location of cancer, age of person, and whether the cancer is in the primary or recurring stages.^22^ For example, a person (<50 y of age), diagnosed with BCC, can undergo a surgical excision known as Mohs surgery. Mohs micrographic surgery removes skin cancer one layer at a time, each time examining these layers under a microscope immediately after removal. Although this procedure allows for minimal scarring by preserving healthy tissue, it is time consuming (3‐4 h to remove a single lesion) and expensive.^23^, ^24^, ^25^ Superficial removal of cancerous tissue can be done with cautery and curettage using a spoon‐like instrument with a sharp edge.^26^ Another surgical procedure that may be used for skin cancer is electrosurgery, which is a procedure that cuts/destroys/cauterizes tissue using a high‐frequency electric current applied locally with a pencil‐shaped metal instrument.^27^

For low‐risk disease or treatment of elderly patients, radiation therapy (external beam radiotherapy or brachytherapy),^28^ topical chemotherapy (5‐fluorouracil),^29^ and cryotherapy (freezing the cancer off)^30^ can provide adequate control of the disease. However, the topical application of 5‐fluorouracil often fails due to the inadequate frequency and/or length of treatment, insufficient drug concentration, and a poor penetration of the cream into the epidermis, which contributes to tumor recurrence.^29^ Hence, personalized/precision medicine has emerged because of its potential to improve the accuracy of tumor targeting and minimize toxicity to normal tissue. The beneficial role of novel‐targeted therapies and the potential use of SNAP‐tag fusion proteins in cutaneous cancers is described hereafter. This review seeks to elaborate more on the applications of these diagnostic and therapeutic treatment modalities.

### 1.2 Targeted drug and molecular therapies in skin cancers

The field of cancer immunotherapy attempts to target and kill cancer cells by manipulating the body's immune system and has been immensely successful for the treatment of skin cancer.^31^ To date, eight drugs have been approved by the US Food and Drug Agency (FDA) for the treatment of metastatic melanoma. These include the chemotherapeutic drug dacarbazine (DTIC) (FDA approved in 1975) and the immunotherapeutic agents such as vermurafenib, ipilimumab (FDA approved 2011), dabrafenib, and trametinib (FDA approved 2013), interleukin‐2 (IL‐2) (FDA approved 1998),^32^, ^33^, ^34^, ^35^ and nivolumab and pembrolizumab (FDA approved 2014)^36^ (Table 1). DTIC is more commonly used as the current standard treatment for metastatic melanoma.^32^, ^33^, ^34^ Until recently, single‐agent chemotherapy using DTIC has produced the best therapeutic outcome, with 5% to 15% of patients responding to the therapy, although less than 2% survive 6 years post treatment.^33^, ^37^ Since the discovery of the BRAF^V600E^ mutation in melanomas, three BRAF inhibitors—vemurafenib, trametinib, and dabrafenib—have been used to stop signals that cause cancer cells to grow and divide. However, these drugs are associated with serious side effects, as highlighted in Table 1.^38^, ^39^

**Table 1:** FDA approved melanoma and BCC treatments and their adverse side effects

Trade Name	Details of Drug	Year of FDA Approval	Type of Cancer	Adverse Effects
DTIC‐Dome (dacarbazine)	Antineoplastic chemotherapy drug.	1975	Melanoma; Hodgkin lymphoma	Respiratory toxicity and dyspnea and hepatic necrosis
Intron (interferon α‐2b)	Biologic response modifier	1995	Malignant melanoma	Flu‐like syndrome, low blood counts, and changes in vision
Aldara (imiquimod)	Immune response modifier	1997	Basal cell carcinoma	Skin reactions, systemic inflammation, and auto‐immune
Proleukin (interleukin‐2)	Antineoplastic biologic response modifier	1998	Metastatic melanoma	Vascular leak syndrome, hypotension, and oliguria
Zelboraf (vermurafenib)	BRAF kinase inhibitor	2011	Melanoma	Skin reactions, photosensitivity, arthralgia, and SCC
Yervoy (ipilimumab)	Monoclonal antibody	2011	Melanoma	Diarrhea, colitis, hypopituitarism, and hypothyroidism
Erbitux (cetuximab)	Monoclonal antibody	2011	Squamous cell carcinoma	Diarrhea, skin toxicity, fatigue, and mucositis
Tafinlar (dabrafenib)	BRAF kinase inhibitor	2013	Metastatic melanoma	Hyperglycemia, hyperkeratosis, and hypophosphatemia
Mekinist (trametinib)	MAP kinase 1 and MAP kinase 2 inhibitors	2013	Malignant melanoma	Skin reactions, cardiomyopathy, and cardiac failure
Opdivo (nivolumab)	Checkpoint inhibitor	2014	Melanoma	Colitis, thrombocytopenia, and lymphopenia
Keytruda (pembrolizumab)	Monoclonal antibody	2014	Metastatic melanoma	Hyperglycemia, hyponatremia, and anemia

As persistent inflammation has emerged as a cardinal hallmark of cancer,^40^ targeting toll‐like receptors (TLRs) is also hypothesized as a plausible potential molecular approach for skin cancer therapies.^41^ Another successful treatment option in patients with melanoma is the use of monoclonal antibodies (mAbs), which are immune checkpoint inhibitors. For example, the monoclonal antibody ipilimumab is directed toward the cytotoxic T‐lymphocyte antigen (CTLA)‐4 and was the first (CTLA)‐4 inhibitor to demonstrate an improved overall survival rate in melanoma patients.^42^ Other mAbs such as nivolumab bind to the programmed‐cell death (PD)‐1 receptor and block interaction with PD‐L1 and PD‐L2 ligands.^43^ This binding releases PD‐1 pathway‐mediated immune responses against tumor cells.^43^ Recently, anti‐programmed cell death‐1 (Anti‐PD‐1) was approved for the treatment of patients with advanced melanoma.^37^ Although PD1 blockers have comparatively better safety, the main concern with PD1 monotherapy is patient response rate (around 30%‐40%).^44^

The pegylated version of interferon α‐2b (PEG‐IFN) has been approved as an adjuvant for surgically resected “high‐risk” melanoma patients.^45^ However, these mAbs are associated with severe side effects, including dermatologic, gastrointestinal, hepatic, endocrine, and, less commonly, inflammatory events.^46^

EGFR (epidermal growth factor receptor) is the first molecular target against which mAbs have been developed for cancer therapy.^47^ Anti‐EGFR mAbs are known to bind to the extracellular domain of EGFR in its inactive state, then compete for receptor binding by occluding the ligand‐binding region, and block ligand‐induced EGFR tyrosine kinase activation.^48^, ^49^ The anti‐EGFR mAb called cetuximab is used in combination with radiotherapy and is considered a promising treatment modality for locally advanced inoperable NMSC.^50^ However, side effects such as a persistent rash are still associated with cetuximab as well as other cutaneous toxicities such as painful fissures in palms and soles and paronychia.^51^ mAbs targeting tumor‐associated cell surface antigens overexpressed on tumor cells but also expressed on normal cells can thus also interact with normal cells.^52^, ^53^ In addition to the related off‐target, nonspecific toxicities, the high proportion of nonhuman sequences eventually incorporated in mAbs is likely to be recognized as “foreign” and therefore induce a host immune response. This can result in reduced efficacy of the mAb, due to increased clearance.^52^, ^53^ Table 1 summarizes the different types of treatments approved for skin cancer and their corresponding adverse side effects.

To provide an improved targeting approach, antibody drug conjugates (ADCs) have been designed, in which a cytotoxic payload is attached to an antibody via a chemical linker.^54^ This is exemplified, for instance, by the novel ADC EV20‐Sap that displayed promising antitumor activity in metastatic melanoma, obtained by chemically coupling the HER‐3 targeting antibody EV20 to the plant toxin saporin.^55^ This conjugate maintained the biological activity of the naked HER‐3 antibody. It binds to melanoma cells with the same affinity as free EV20 and eliminated cancer cells, upon internalization with IC50 values in the range of 0.15nM to 20nM. This attests to its powerful specificity and target‐dependent cytotoxic activity. Furthermore, in a murine melanoma model, EV20‐Sap treatment led to a significant reduction of pulmonary metastasis.^55^

Despite the therapeutic efficacy of ADCs, their major challenges were size and heterogeneity.^56^, ^57^ The large size of mAbs (150 kDa) might exhibit relatively limited tissue penetration and is prone to nonspecific binding owing to their Fc domain.^58^ Furthermore, the cytotoxic agents in ADCs are typically conjugated randomly to the antibodies, using either the reduced sulfhydryl groups of cysteine residues or the amino groups of lysine side chains. This generates heterogeneous ADC populations with variable drug to antibody ratios (DAR) that results in reduced efficacy and unpredictable pharmacokinetic profiles.^56^, ^57^ While one might presume that high‐affinity binding is ideal, several studies have shown that very high affinities might be suboptimal for therapeutic antibodies to penetrate deep into solid tumors.^59^, ^60^ This results in rapid and tight binding to the outer surface of a tumor and reduced numbers of antibodies diffusing to the core of the tumor. This is because rate of diffusion is approximately inversely proportional to the cube root of molecular weight.^60^, ^61^ Thus, the intended effects of the mAb would not be universal to all tumor cells.^60^, ^62^

Many nanoparticle‐based drug delivery systems have been approved by the FDA and are currently undergoing clinical trials for skin cancer therapy.^63^ It has been shown that delivering the chemotherapeutic agent doxorubicin by gold nanoparticles was very effective against a melanoma cell line.^64^ Lo Prete et al applied a cholesterol‐rich nanoemulsion to deliver etoposide in a mouse model of melanoma.^65^ The nanoemulsion delivery was associated with decreased side effects, increasing maximum tolerated dose fivefold and increased inhibition of tumor growth by concentrating etoposide at the tumor site (a fourfold higher concentration in tumor than with free etoposide).^65^ Nonetheless, nanoparticles as efficient drug delivery systems are hindered by incomplete toxicological assessment, low drug‐loading capacities, difficulty in scale‐up production, and low stability.^66^

Natural compounds have been suggested for use alone^67^, ^68^ or in combination with photodynamic therapy (PDT)^69^ in the treatment of skin cancer. PDT is a treatment modality that uses an effector molecule called a photosensitizer (PS), followed by local illumination with visible light of specific wavelength(s). When a PS is exposed to a specific wavelength of light, it produces reactive oxygen species (ROS) that induce apoptosis of cancerous lesions.^70^ To date, targeting cancer cells using PDT has relied on the passive accumulation of PS in tumor tissues, which might not lead to optimal dosage of PS, thus leading to the application of relatively high dosage of PS within the tumor.^71^ As a result, PDT may damage healthy tissues, by causing prolonged skin photosensitivity.^72^ Efforts to bypass this lack of specificity have focused on the identification of specific cancer biomarkers, drug conjugates, and resistant mechanisms contributing to cancer survival after therapeutic treatments. The addition of SNAP‐tag technology to skin cancer management potentially presents a more structurally reliable method for conjugation and delivery of photosensitizer or cytotoxic payload for targeted cancer chemotherapeutic purposes, as discussed below.

### 1.3 Targeted drug conjugation and SNAP‐tag technology

**Figure 2:**
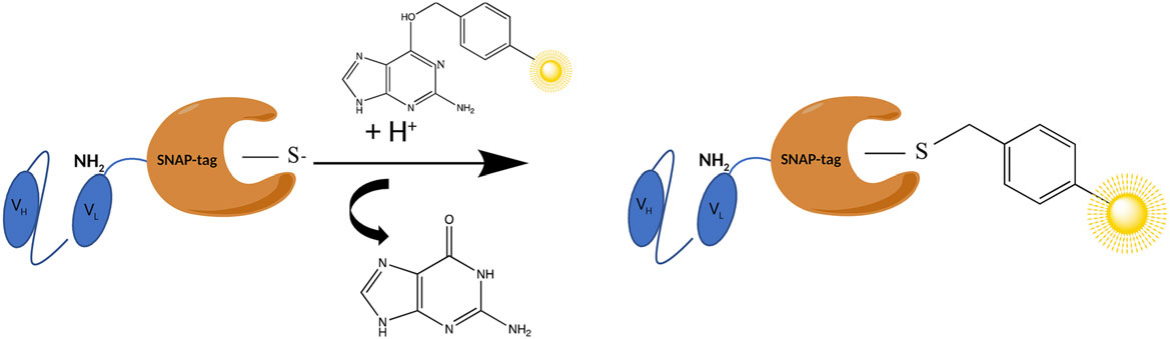
The autocatalytic reaction of scFv‐SNAP genetically fused to the amino terminus of the VL chain of the scFv and conjugated to a BG‐modified photosensitizer (in yellow)

SNAP‐tag is a mutant form of the enzyme O6‐alkyguanine‐DNA alkyltransferase, used as a tag for self‐labeling with modified O(6) benzylguanine (BG) substrates via an irreversible transfer of an alkyl group to a cysteine residue within its active site (Figure 2).^73^ Different BG‐modified effector molecules, eg, photosensitizers, toxins, or fluorophores, can be conjugated to SNAP‐tag in a site‐specific and selective manner for diagnostic or therapeutic treatment of cancer, without affecting the activity of the recombinant ligand.^74^, ^75^, ^76^ Fusing SNAP‐tag to recombinant antibodies by protein engineering provides a new antibody format that is designed to overcome the problems of nonspecific targeting and heterogeneity; the efficient directed, covalent conjugation is provided by an autocatalytic reaction (Figure 2) under physiological conditions, providing a 1:1 stoichiometry between recombinant SNAP‐tag–based antibody fusion protein and BG‐modified small synthetic substrate.^73^

Besides the fact that the recombinant expression of antibody genes is difficult because of their large size, the usage of whole immunoglobulins (IgGs) can cause unwanted side effects mediated by the constant (Fc) region of the antibody.^60^ To overcome this challenge, SNAP‐tag fusion proteins are engineered with antibody fragments called single‐chain variable fragments (scFvs), which are formed by the tandem arrangements of the heavy (V_H_) and light chain (V_L_) domains joined by a flexible serine/threonine linker (Figure 2).^77^, ^78^ There is no preferential orientation of one domain to the other, and V_H_‐L‐V_L_ and V_L_‐L‐V_H_ constructs are likely equivalent. Most scFv fragments are generated using a 15‐amino acid residue linker of composition (Gly_4_Ser)_3._ 79 The biological effects of the scFv can be enhanced by (1) reducing the length of the linker, resulting in paired scFvs that bind to one another through complementary regions to form bivalent molecules called diabodies, (2) further shortening of linker to form trimers/tetramers, or (3) complementary scFvs produced as a single chain called tandem scFvs.^77^ The small size of the scFv (27 kDa) allows for better clearance from the body, better tissue/tumor penetration, and simple and straightforward production in bacterial cell systems vs mammalian cells.^74^, ^76^, ^80^ The construct also produces a high tumor to background ratio with high visualization and a low nonspecific background signal.^81^, ^82^, ^83^, ^84^ By identifying tumor‐specific antigens (TAAs) for melanoma, BCC, or SCC and targeting them with an advanced recombinant SNAP‐tag antibody‐labeling technology, tumors can be screened prior to therapy and the appropriate treatment modality implemented.^85^ One such treatment modality that shows promising potential with the use of SNAP‐tag technology is photoimmunotherapy (PIT).

**Figure 3:**
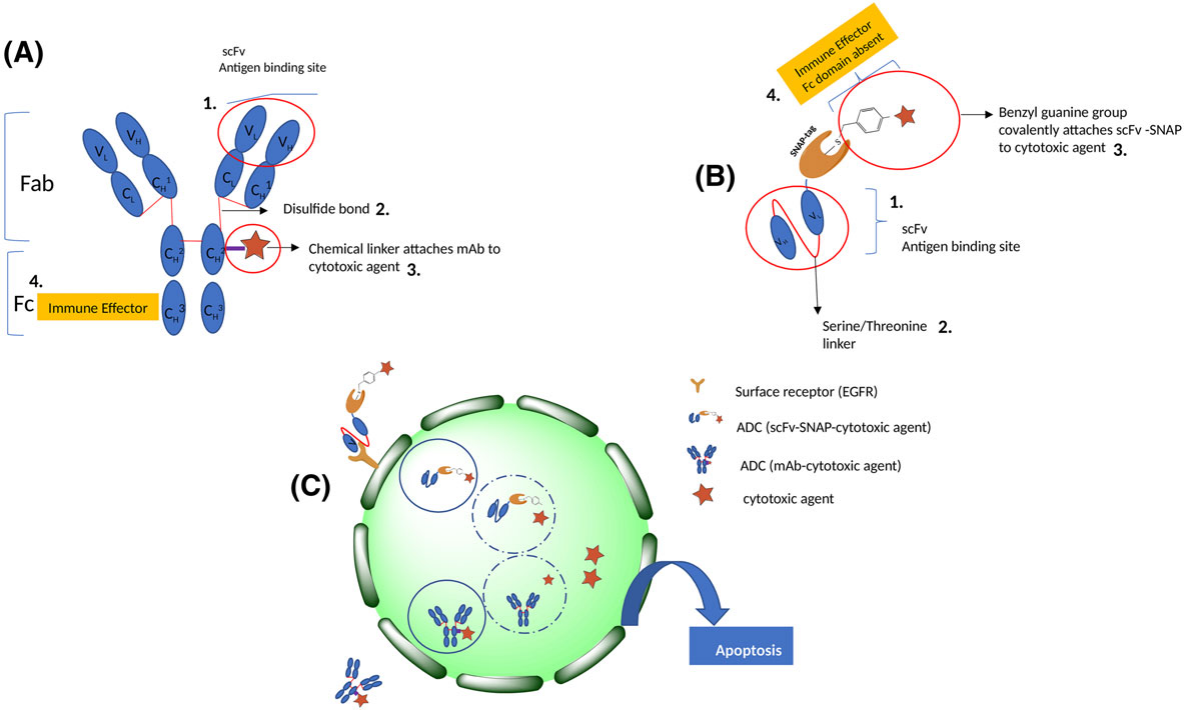
Structure of two types of ADCs: A, an immunoglobulin (IgG) with variable (V) and constant (C) regions conjugated to a cytotoxic agent and, B, a single‐chain variable fragment (scFv) attached to SNAP and conjugated to a benzylguanine modified cytotoxic agent. C, Mechanisms of uptake and internalization common to both types of ADCs

A SNAP fusion protein is a type of ADC that exhibits similarities and differences to conventional ADCs. The following similarities between the both are clear from Figure 3: (1) Both the monoclonal antibody and the SNAP fusion protein share similar features in structure in that they both contain variable light and heavy chains (scFvs) with complementarity determining regions (CDRs) that constitute the antigen‐binding region of the antibody (Figure 3; A1 & B1). This corresponding paratope is specific for tumor‐associated epitopes that are restricted in their expression on healthy cells. (2) Mechanisms of receptor‐mediated uptake and internalization are common to both types of ADCs within a tumor cell, where apoptosis is induced by the release of the cytotoxic agent into the cytosol (Figure 3C).

The following differences between conventional ADCs and SNAP fusion proteins are clear from Figure 3: (1) Disulfide bonds link the heavy and light chains of the IgG, while a (Gly_4_Ser)_3_ linker connects heavy and light chains in an scFv (Figure 3; A2 & B2). (2) The method of conjugation of a drug/cytotoxic agent to the antibody within a conventional ADC differs from that of a SNAP fusion protein. For SNAP fusion proteins, a chemical linker is attached to the cytotoxic drug via the lysine or cysteine amino acid side chains on the mAb, creating a variety of possible conjugation sites for the drug (DAR > 1), and this creates heterogeneous constructs with unpredictable pharmacokinetic profiles, off‐target side effects and a relatively low maximum tolerated dose.^86^ The type of linker (cleavable or noncleavable) also impacts on the efficacy of the ADC. For example, these linkers facilitate premature spontaneous drug release, which damages normal tissues.^87^ SNAP‐tag is genetically fused to the scFv and conjugated to the cytotoxic agent via an autocatalytically generated covalent bond through the cysteine residue of the enzymes catalytic site with the benzylguanine‐modified agent to generate a homogenous construct with a higher therapeutic index.^73^, ^75^ This creates a single specific site for conjugation and allows an optimal 1:1 stoichiometry, which does not affect the activity of the recombinant ligand and overcomes the challenges of current ADC conjugation strategies that are highlighted above^88^ (Figure 3; A3 & B3). (3) The Fc region is the domain that determines the effector function of the IgG, that is, how it engages with specific cell receptors or other defense proteins, eg, B lymphocytes, in order to elicit an immune response to cause cell death.^89^ The Fc region is not present in the antibody part of SNAP fusion proteins, such that the cytotoxic agent alone elicits the effector function by lysosomal release into the cytosol to induce apoptosis (Figure 3; A4, B4 & C).

#### 1.3.1 Photoimmunotherapy

Photoimmunotherapy (PIT) consists of conjugating a photosensitizer to a tumor cell–specific mAb.^90^, ^91^ Research show that PIT has been influential in treating melanoma with laser light. Naylor et al combined laser treatment with imiquimod to treat melanoma. The laser light devitalized the tumor and converted the cells from a viable tumor to antigenic materials for the immune system to respond to. Continued therapy with imiquimod after laser therapy ensured that the devitalized tumor was engulfed and processed by recruited dendritic cells.^92^ In 2010, another in situ PIT study was performed on patients with metastatic melanoma.^93^ The components of PIT included the local application of imiquimod irradiated with laser light. Eleven patients received this treatment modality in one or multiple 6‐week treatment cycles, and a 12‐month overall survival rate of 70% with no toxic side effects was observed.^93^ In 2017, Naylor et al used a novel immunological approach for treatment of metastatic cancers, called laser immunotherapy, in combination with the check point inhibitor ipilimumab, to treat melanoma.^94^ It was observed that after laser immunotherapy on one patient, cutaneous melanoma in the head and neck completely disappeared.^94^ The patient was then administered one course of ipilimumab, 3 months after laser treatment, and all tumor nodules in the lung decreased. The patient remained tumor free for 1 year.^94^ This highlights the efficacy of combination treatment in enhancing therapeutic effects in melanoma treatment.

Another newly developed cell‐selective cancer therapy is near‐infrared photoimmunotherapy (NIR‐PIT). Its promising potential in skin therapy is attributed to reduced photon scattering, light absorption, and auto‐fluorescence, as well as increased light penetration into tissue, as compared with PDT.^95^ NIR‐PIT can target a broad array of cancer‐specific target molecules including the proteins EGFR, HER2, PSMA, CD25, CEA, mesothelin, GPC3, and CD20.^96^ Since NIR‐PIT can selectively kill off target cells, it can be used to eliminate cancer stem cells displaying markers such as CD44 and CD133, as was demonstrated for breast cancer and glioblastoma stem cells.^96^ This creates the possibility of using this option to target antigens associated with melanoma or BCC.

EpCAM (CD 326) is a human transmembrane glycoprotein located on the cell membrane and within the cytoplasm of all non‐squamous epithelial cells.^97^ Previous studies have shown that anti‐EpCAM antibody Ber‐EP4 is a sensitive marker of basal cell carcinoma; however, it fails to stain cutaneous squamous cell carcinoma.^98^, ^99^ Chondroitin sulfate proteoglycan 4 (CSPG4), also known as melanoma chondroitin sulfate proteoglycan (MCSP), is a membrane‐bound proteoglycan and was initially characterized on the surface of melanoma cells.^100^, ^101^ Targeting CSPG4 was also shown to be clinically relevant by an increase in survival of melanoma patients who received CSPG4 mimics as a form of active specific immunotherapy.^102^ Targeting CSPG4 also inhibited the growth and recurrence of melanoma in a human melanoma xenograft model.^103^

Two photosensitizers (PS) that are currently used in NIR‐PIT are IR700 and hypericin. IR700 is a promising PS that, besides having no off‐target effects, possesses ideal properties such as high purity, photostability, and a strong absorption peak close to 700 nm allowing improved light penetration into tissues.^90^, ^104^, ^105^, ^106^, ^107^ Recently, the IR700 fluorophore was conjugated to a scFv fragment against three overexpressed cancer antigens, ie, the EGFR, EpCAM, and CSPG4, using SNAP‐tag technology.^76^, ^88^ In vitro success of this therapeutic approach in killing melanoma cells was attributed to the scFv‐425 (EGFR) targeted effect, as well as the nontoxic effect of free IR700 even after irradiation.

Hypericin (HYP) has been shown to be an effective second‐generation PS. Hypericin is a natural photosensitizer, biosynthesized within the dark glands of the petals and leaves of the St John's Wort plant (Hypericum perforatum).^108^, ^109^ It belongs to the chemical class of naphtodianthrones and can be chemically synthesized through conversion of emodin to hypericin using Hyp‐1 enzyme, yielding approximately 84.6% efficient conversion when overexpressed in *Escherichia coli*.^110^, ^111^ Hypericin‐based PDT treatment was shown to be effectively cytotoxic to metastatic melanoma through the localization of HYP in melanosomes.^112^, ^113^, ^114^ Another study showed that hypericin can inhibit the growth of SCC tumors in culture and can reduce tumor size in mice in the complete absence of light.^115^ Recently, an attempt to circumvent chemoresistance was made by Biteghe and Davids, who by combining DTIC with hypericin were able to overcome this resistance due to the genotoxic effect by DTIC and the oxidative stress induced by HYP‐PDT.^116^ Optical imaging methods have also seen widespread application in skin cancer diagnostics as they are noninvasive, with fast response times, and are potentially sensitive to biochemical and structural changes presented in skin cancer development.^117^, ^118^

### 1.4 Optical imaging methods in cancer diagnosis

For skin cancer diagnostics, the primary optical imaging techniques used are widefield imaging, optical spectroscopy, and microscopy imaging.^119^ Widefield imaging allows the examination of large areas and has the potential of improving detection of hidden lesions, margin delimitation, and also guide biopsy site determination.^120^ A major advantage of the widefield microscope is the low cost, simplicity, and flexibility of the system.^121^ In contrast, some disadvantages of widefield microscopy include low image resolution, potential for shading artifacts due to uneven illumination, and the alignment of different cameras to ensure pixel registration when using multiple indicators.^122^ Microscopy imaging has the main advantage of the evaluation of the tissue characteristics at cellular level, but only a small fraction of the lesion volume is interrogated.^123^

Optical spectroscopy presents more detailed information on tissue composition than widefield microscopy, as the light intensity for each collected emission wavelength is correlated to specific biomolecules.^124^ Raman spectroscopy has gained considerable interest in disease diagnosis, particularly cancer, because of its ability to provide molecular specific information about tissues. Each Raman spectral peak can be associated with specific vibrations in molecular bonds.^125^, ^126^ Thus, this technique provides biochemical information about a sample, including conformations and concentrations of constituents.^127^ Different forms of Raman spectroscopy have evolved to meet requirements in a specific biological application. However, due to long integration times, bulky instrumentation, high excitation intensities, and mutagenicity of the UV light, Raman has limitations for in vivo use.^126^ Thus, NIR dispersive Raman spectroscopy, in which NIR excitation minimizes fluorescence and absorption by tissue, has been the technique of choice for in vivo applications.^126^

Fluorescence imaging is another optical method based on the use of fluorophores, which are compounds that can emit light after absorption of the appropriate wavelength light.^128^ NIR fluorescent probes are advantageous for in vivo imaging because of minimum photodamage to biological samples, deep tissue penetration, and minimum interference from background autofluoresence by biomolecules in living systems.^95^ SNAP‐tag technology provides a unique antibody format that allows for site‐specific conjugation of organic/inorganic fluorophores or fluorescence nanoparticles in the NIR spectral region. For accurate imaging, the nanoparticles are conjugated with targeting ligands and/or constructed as off‐on probes. Polyglycerol doxorubicin was conjugated to EGFR‐specific (scFv‐425)‐SNAP‐tag fusion proteins for targeted delivery to different cell lines. These SNAP‐tag–conjugated nanoparticles showed increased specificity, no off‐target internalization, and accumulation and EGFR concentration‐dependent toxicity, warranting further in vivo studies of scFv‐SNAP fusion proteins with multifunctional polyglycerol.^129^ Petershans et al developed a method for protein immobilization onto modified CdSe/ZnS quantum dot surfaces using simple SNAP‐tag methodology.^130^ Mazzucchelli et al designed a SNAP fusion protein, which was irreversibly immobilized on magnetofluorescent nanoparticles through the recognition between SNAP and a pegylated O_6_‐alkylguanine derivative. The targeting efficiency of the resulting nanoparticle against HER2‐positive breast cancer cells was assessed by flow cytometry and immunofluorescence.^131^ In addition, an epidermal growth factor–based nanoprobe (EGF‐NP) for in vivo optical imaging of epidermal growth factor receptor (EGFR) was developed. The NIR fluorophore (Cy5.5) and quencher (BHQ‐3) was sequentially conjugated to EGF (6.2 kDa) compared with EGFR antibody (150 kDa).^132^ The self‐quenched EGF‐NP exhibited great specificity to EGFR and rapidly internalized into the cells, as monitored by time‐lapse imaging.^132^ Importantly, the self‐quenched EGF‐NP boosted strong fluorescence signals upon EGFR‐targeted uptake into EGFR‐expressing cells, followed by lysosomal degradation, as confirmed by lysosomal marker cell imaging.^132^

In a study by Gong et al, an NIR fluorescent SNAP‐tag substrate BG‐800 was synthesized by conjugating an IRDye 800CW to the benzyl‐guanine amino group (BG‐NH_2_) of the protein tag.^84^ Because BG‐800 was cell impermeable, the SNAP_f_‐ADRβ2 fusion protein was used in such a way that ADRβ2 directed the localization of SNAP_f_ fusion protein to the cell membrane. BG‐800 reacted with SNAP_f_‐ADRβ2 in both cell lysate and live cell culture.^84^ The tumor expressing SNAP_f_‐ADRβ2 was then visualized using BG‐800 conjugated to the IRDye 800CW. SNAP(f) is a fast‐labeling variant of SNAP‐tag showing an improved reaction with benzylguanine (BG)‐modified synthetic substrate, leading to a faster covalent attachment of substrate to the SNAP(f). This property makes SNAP(f) a valuable tool for imaging applications. SNAP(f)‐beta‐2 adrenergic receptor (SNAP(f)‐ADRβ2) fusion protein was created with the ADRβ2 portion of the protein directing the localization of the protein to the cell membrane.^84^

Rapid optical imaging of EGF receptor expression with a single‐chain antibody SNAP‐tag fusion protein was also studied. EGF receptors (which is a member of the receptor tyrosine kinase [RTK] family) are usually overexpressed in cancer, even though healthy cells also express them.^133^ The EGFR‐specific scFv fusion protein 425‐SNAP was labeled with the NIR dye BG‐747, and its accumulation, specificity, and kinetics were monitored using NIR fluorescence imaging in a subcutaneous pancreatic carcinoma xenograft model.^74^ The 425 (scFv) SNAP fusion protein accumulated rapidly and specifically at the tumor site. Its small size allowed efficient renal clearance and a high tumor to background ratio (TBR).^74^

The SNAP‐tag can also be combined with other protein tags, such as HaloTag,^134^ or other reporter gene systems that use fluorescent substrates, such as β‐galactosidase/DDAOG system,^135^ to create multiplexed imaging systems. A second version of AGT‐based tag named CLIP‐tag reacts specifically with benzylcytosine (BC) derivatives.^136^ Because SNAP‐tag and CLIP‐tag only react with their specific substrates, they could be used simultaneously for dual‐color fluorescence imaging.^137^ For example, the nonspecific blood flow tracer indocyanine green (ICG) was successfully used to visualize regional lymphatic flow from cancer lesions and identified sentinel lymph nodes in humans.^137^ Simultaneous but separate visualization of different lymphatic drainages was made possible by fluorescent agents with multiple colors.^137^ The clinical use of NIR fluorescence imaging for sentinel lymphatic mapping was first reported by Kitai et al in 18 breast cancer patients.^138^ They injected 25 mg of ICG near the areola of breast cancer patients and successfully visualized the draining lymphatics in all patients and localized the sentinel lymph nodes in 17 of 18 patients.^138^ Following this study, additional clinical studies have confirmed the utility of NIR sentinel mapping in melanoma.^139^ Tumor detection with NIR fluorescence during a surgical procedure has been performed in several tumor types, with application in melanoma using ICG.^140^ The subsequent conjugation of ICG to SNAP‐tag thus creates new possibilities for image guided surgery in melanoma patients in the future.

**Figure 4:**
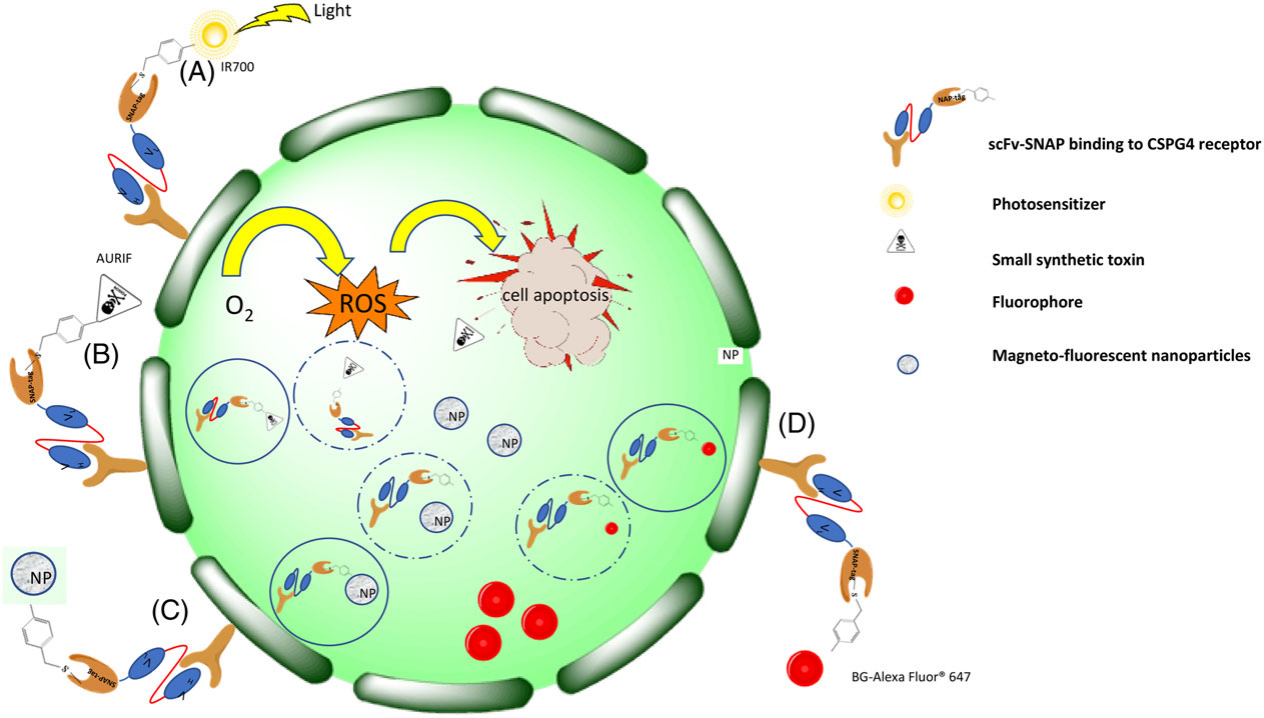
A summary of the diagnostic and therapeutic applications of SNAP‐tag fusion proteins on a tumor cell expressing the extracellular receptor CSPG4. A, scFv targets the fusion protein to the surface receptor on the tumor cell by photoimmunotherapy. B, Auristatin F‐SNAP‐tag conjugate gets internalized and released into the cytosol where it induces apoptosis. C, Magnetofluorescent nanoparticles and, D, fluorophores enter the cell by receptor‐mediated uptake and accumulate within the tumor and allow for optical detection

As shown in Figure 4A, the scFv targets the fusion protein to the surface receptor on the tumor, cell and the conjugated photosensitizer (IR 700) is activated by a specific wavelength of light (500‐700 nm). The energy‐enriched photosensitizer releases the extra energy to its surroundings and returns to the ground state. Singlet O_2_ is converted to reactive oxygen species (ROS), which induces apoptosis/necrosis of tumor cells.^88^ This application is referred to as photoimmunotherapy; in Figure 4B, auristatin F (AURIF) (microtubule destabilizer) conjugated to the SNAP‐tag antibody fusion protein gets internalized and released into the cytosol where it induces apoptosis,^141^ referred to as ADC therapy. In Figure 4; C & D, fluorophores and magnetofluorescent nanoparticles enter the cell by receptor‐mediated uptake and used for optical imaging.^131^, ^142^ After their release, their corresponding signals accumulate within the tumor and allow for optical detection. These are a few examples that prove the versatility of SNAP‐tag technology, which depending on the type of the BG modified substrate conjugated will either elicit signal accumulation for diagnosis^88^, ^131^, ^142^ or induce apoptosis to eliminate cancer cells.^141^, ^143^

## 2 CONCLUSION

In the era of precision medicine, SNAP‐tag technology is a potentially promising molecular targeting approach for early diagnosis and treatment of skin cancer, which has a high burden globally. In this review, we have identified and discussed the prospects for the use of SNAP‐tag for targeted therapy of skin cancers, as well as some of its potential advantages over currently available conventional skin cancer treatment options.

Not least, the use of SNAP‐tag technology in combination with other recently emerging 'omics‐based technologies can potentially offer a treasure trove of targeted diagnostic, prognostic, and therapeutic options for the management of skin cancers in a systems‐oriented manner.

## CONFLICTS OF INTEREST

The authors declare that they have no conflict of interest.

## AUTHOR CONTRIBUTIONS

Conceptualization: Nonhlanhla Patience Khumalo, Stefan Barth

Methodology: Henry Ademola Adeola, Eden Rebecca Padayachee, Jennifer Catherine Van Wyk, Stefan Barth

Resources: Eden Rebecca Padayache, Henry Ademola Adeola, Stefan Barth, Nonhlanhla Patience Khumalo

Visualization: Henry Ademola Adeola, Eden Rebecca Padayachee

Writing – original draft preparation: Eden Rebecca Padayachee, Henry Ademola Adeola, Jennifer Catherine Van Wyk, Fleury Augustine Nsole Biteghe, Shivan Chetty

Writing – review and editing: Henry Ademola Adeola, Eden Rebecca Padayachee, Stefan Barth, Nonhlanhla Patience Khumalo
